# Adaptive immunity at the crossroads of autophagy and metabolism

**DOI:** 10.1038/s41423-021-00662-3

**Published:** 2021-03-30

**Authors:** Shree Padma Metur, Daniel J. Klionsky

**Affiliations:** University of Michigan, Life Sciences Institute and Department of Molecular, Cellular and Developmental Biology, Ann Arbor, MI USA

**Keywords:** Immunology, Lysosome, Macroautophagy, Stress, Biological sciences, Macroautophagy, Lymphocyte activation

## Abstract

The function of lymphocytes is dependent on their plasticity, particularly their adaptation to energy availability and environmental stress, and their protein synthesis machinery. Lymphocytes are constantly under metabolic stress, and macroautophagy/autophagy is the primary metabolic pathway that helps cells overcome stressors. The intrinsic role of autophagy in regulating the metabolism of adaptive immune cells has recently gained increasing attention. In this review, we summarize and discuss the versatile roles of autophagy in regulating cellular metabolism and the implications of autophagy for immune cell function and fate, especially for T and B lymphocytes.

## Introduction

Multicellular organisms have developed remarkable strategies to overcome threats posed by a myriad of pathogens. The adaptive immune system includes specialized cellular subpopulations that can distinguish various pathogens by recognizing unique molecular markers or antigens. The adaptive immune response is a result of the coordinated interplay between antigen-presenting cells and T and B lymphocytes. Effector cells emigrating from the spleen and lymph nodes travel to the sites of infection in harsh nutrient-deprived environments to exert their immune functions. A defining feature of the adaptive immune system is its ability to elicit adequate responses against unique antigens and generate immunological memory for long-term protection against secondary infections. A key bottleneck in the entire process, from lymphocyte development and differentiation to memory generation, is the bio energetically intense mechanisms that require large amounts of metabolites for the proliferation and differentiation of immune cells. However, immune cells lack significant nutrients stores and therefore must dramatically enhance the uptake of substrates such as glucose, amino acids, and fatty acids from the microenvironment. The function of adaptive immune cells is, therefore, bio energetically supported by cellular metabolism, and lymphocytes utilize distinct metabolic substrates and pathways to fuel their effector functions in naïve, activated, and memory states.

In nutrient-deprived microenvironments, an important stress response pathway called macroautophagy/autophagy enables metabolic adaptation by ensuring the cellular availability of the biomolecules required for lymphocyte survival and function. Furthermore, lymphocytes can accumulate deleterious products and a surplus of organelles when engaged in proliferation and differentiation programs. Autophagy is a homeostatic degradation pathway that involves the removal of damaged cellular components, and, for most cells, it is the only mechanism by which entire organelles are eliminated. Therefore, the autophagy pathway is crucial for maintaining the cellular and metabolic homeostasis of lymphocytes.

Several studies over the past decade have highlighted that the various stages of lymphocyte cell fate, from development to differentiation and acquisition of effector function, are intimately linked to dynamic changes in cellular metabolism and the autophagy pathway. Here, we discuss how the autophagy pathway and autophagy proteins shape the adaptive immune response at the cellular level in conjunction with the internal metabolic state. Importantly, we discuss the metabolic control of autophagy in lymphocyte lineage specification and attainment of functional competency. Further, we focus on how cellular metabolism and autophagy integrate with immune suppression and memory generation.

## Molecular mechanisms of autophagy

Macroautophagy is an evolutionarily conserved nutrient recycling pathway that delivers cytoplasmic components to the lytic compartment of a cell. This process can be divided into nonselective/bulk autophagy and selective autophagy^1^. In bulk autophagy, the cargo is randomly selected and sequestered in a cup-shaped structure called a phagophore. The expansion and closure of a phagophore results in the engulfment of part of the cytoplasm within a double-membrane structure called an autophagosome, which subsequently fuses with a lysosome where lumenal components are degraded; the resulting breakdown products are then released back into the cytosol for reuse. This process occurs at a low basal level in virtually all cells. However, autophagy is upregulated in response to metabolic stress. Autophagy, therefore, helps maintain metabolic homeostasis by generating macromolecules that fuel anabolism and enable cell survival^2,3^.

Macroautophagy is the best-characterized type of autophagy and involves five major steps: initiation, nucleation, expansion, fusion, and degradation-recycling^4^. The initiation of the double-membrane phagophore begins with the phosphorylation of various components of the PIK3C3/VPS34 kinase complex by the ULK kinase complex comprised of ULK1/ULK2, RB1CC1/FIP200, ATG13, and ATG101. This phosphorylation results in the activation of the lipid kinase PIK3C3/VPS34 and the production of local pools of phosphatidylinositol-3-phosphate (PtdIns3P) needed for the nucleation of the phagophore^5^. The ATG9 trafficking system, composed of ATG2, WDR45/WIPI4, and the transmembrane protein ATG9A in conjunction with lipids channeled from the ER, supplies membrane precursors to meet the high demand for the membrane required for autophagosome biogenesis^6–9^. Following phagophore nucleation, two essential ubiquitin-like conjugation systems are activated for membrane expansion and fusion. The two systems function to covalently conjugate Atg8-family proteins (i.e., the MAP1LC3/LC3 and GABARAP subfamilies) to the expanding phagophore. First, the E1-like enzyme ATG7 and the E2-like enzyme ATG10 conjugate the ubiquitin-like protein ATG12 to ATG5. This complex subsequently binds to ATG16L1 and acts as an E3 enzyme for the conjugation of Atg8-family proteins to the membrane-resident lipid phosphatidylethanolamine as facilitated by the E1 enzyme ATG7 and E2 enzyme ATG3. Lipidation of Atg8-family proteins drives phagophore expansion and facilitates the recognition of specific cargo via interaction with receptors^10,11^. Upon completion of phagophore expansion and closure, the resulting double-membraned autophagosome topologically separates the autophagic cargo from the cytoplasm. Following the dissociation of the autophagic machinery from its surface, the outer membrane of the autophagosome fuses with a lysosome to form an autolysosome. Subsequently, the inner membrane and its enclosed contents are exposed to lysosomal resident hydrolases and degraded to generate simple metabolites, which are released into the cytoplasm via transporters, and subsequently reused.

## Selective autophagy

Specific targeting of superfluous organelles to the lytic compartment by autophagic mechanisms facilitates cellular remodeling to accommodate metabolic state switching^12^. The selectivity of autophagy is conferred by receptors that tether cargo to lipidated LC3. There are three types of receptors with functions in autophagy: first, receptors that specifically recognize and directly interact with a cargo protein; second, receptors that recognize ubiquitination of cargo; and third, receptors that are components, typically integral membrane proteins, of the cargo^13^. Subsequently, the cargo is engulfed by the phagophore and targeted for lysosomal degradation. Different forms of selective autophagy are named on the basis of the cargo source, such as mitophagy, reticulophagy, pexophagy, etc^1^.

### Mitophagy

Selective clearance of damaged or superfluous mitochondria by the canonical autophagy machinery is termed mitophagy^14^. This process limits the generation of reactive oxygen species (ROS) and maintains mitochondrial integrity and oxygen homeostasis. PINK1 (PTEN induced kinase 1)- and PRKN/Parkin (parkin RBR E3 ubiquitin-protein ligase)-mediated mitophagy involves a well-characterized ubiquitin-dependent pathway. In addition, there is a PINK1-PRKN-independent mitophagy pathway. Mitophagy receptors such as SQSTM1/p62, CALCOCO2/NDP52, and OPTN recruit LC3 to ubiquitinated mitochondria via LC3-interacting region motifs^14^.

TFAM (transcription factor A, mitochondrial) regulates mitochondrial biogenesis. A recent report indicated that T cells with dysfunctional mitochondria associated with TFAM deficiency display signs of accelerated senescence, including the accumulation of circulating cytokines; this phenotype of chronic inflammation is referred to as “inflammaging”^15^. Although no direct connection between autophagy and inflammaging has been established to date, it is reasonable to speculate that defects in mitophagy and the associated accumulation of damaged mitochondria might contribute to a prolonged state of inflammation. In addition, autophagy normally acts to suppress various cytokines and proinflammatory complexes, such as the NLRP3 inflammasome^16^.

### Reticulophagy

The endoplasmic reticulum undergoes dynamic changes in morphology, composition, and functionality. For the efficient and continual performance of the ER, remodeling the network in response to stress is necessary, and autophagy is a key player in remodeling this organelle. Following autophagy induction, autophagy machinery is recruited to the ER, where portions of the ER are sequestered within autophagosomes via the recognition of various reticulophagy receptors, such as SEC61, RTN3, and TEX264. The selective degradation of ER portions limits stress and prevents apoptosis^17^.

## Autophagy and immunity

Autophagy plays a crucial role in initiating and supporting several processes in both innate and adaptive immunity. ATG proteins directly contribute to pathogen clearance through selective autophagy of microorganisms, coordinated response with pattern recognition receptors, inflammasome formation, antigen presentation, and LC3-associated phagocytosis^18^. In adaptive immunity, autophagy modulates antigen processing and presentation and regulates the development of lymphocytes^19^. Regulation of the organelle content of lymphocytes, especially mitochondria, by autophagy is crucial during differentiation. Further, several studies have shown that mitophagy maintains immune cell homeostasis by restricting inflammatory cytokine secretion. Moreover, mitophagy is also involved in suppressing mitochondrial antigen presentation, a process by which mitochondrial antigens are presented on the cell surface to trigger an immune response^20^. In addition, autophagy controls ER shape and function in lymphocytes to maintain homeostasis^21^. Autophagy plays an important role in the metabolic switching required for immunosuppression and memory generation. In this review, we discuss the interplay of autophagy and cellular metabolism in controlling adaptive immunity.

## Lymphocyte metabolism

All cells, including quiescent, proliferating, and differentiating immune cells, require sufficient energy to support their basic cellular functions. Glycolysis, the TCA cycle, and oxidative phosphorylation (OXPHOS) form the core metabolic pathways that function to meet the bioenergetic demands of a cell. Glucose, fatty acids, and glutamine can fuel OXPHOS, whereas cells are directly dependent on glucose as the carbon source for generating ATP via glycolysis. Metabolic flexibility plays a major role in interchanging carbons from various macromolecules and thus ensuring bioenergetic adaptation to changing nutrient and oxygen levels^22–24^. However, metabolism is not limited to merely biosynthesis and metabolite degradation. Abundant evidence suggests that immune cell function and fate are intricately tied together with metabolism. Differentiation programs that lead to different subsets of immune cells adopt unique metabolic states that are closely tied to transcriptional and translational regulatory landscapes^25^. One of the earliest studies on the importance of cellular metabolism in immune cells was conducted by Otto Warburg, who showed that stimulated leukocytes prefer glycolysis over OXPHOS^26^. Furthermore, his quantitative investigations of cancer metabolism helped establish that the transition from aerobic OXPHOS to glycolysis in the presence of oxygen is a hallmark of many cancer cells^27^. This metabolic reprogramming of highly proliferative cells termed aerobic glycolysis or the Warburg effect is also a hallmark of lymphocyte activation and has been the focus of several studies, especially in the last decade^28^.

Different subpopulations of lymphocytes arise from a common lymphoid progenitor derived from hematopoietic stem cells. Following lineage commitment, these cells show diverse functionality and localizations. T lymphocytes mature in the thymus, whereas B lymphocytes arise in the bone marrow^29^. Following the development in the primary lymphoid organ, these cells travel to secondary lymphoid organs such as lymph nodes and the spleen, where they are presented with either circulating antigens or pathogen-activated antigen-presenting cells and thus attain maturity. Naïve lymphocytes are quiescent with low transcriptional and metabolic activity. Activation, which involves the engagement of specific surface proteins on lymphocytes such as T cell and B cell receptors (BCRs), results in dramatic cellular changes^30^. Lymphocyte activation is accompanied by several phenotypic alterations, such as clonal expansion and an increase in metabolic demands of enhanced proliferation and differentiation. As indicated above, for metabolism and functional competency, cellular bioenergetic demands must be met. In support of these requirements, activated lymphocytes enhance glucose uptake and subsequent metabolism. Although OXPHOS is highly efficient in converting glucose to ATP, it provides very little energy for macromolecular synthesis. Conversely, glycolysis along with glutamine metabolism can provide sufficient ATP and the intermediate metabolites that are needed for lymphocyte activation and effective immune response. Therefore, the metabolic flexibility conferred by the transition from OXPHOS to glycolysis underpins this activation program by providing the necessary intermediate metabolites. In addition, the burden of increased metabolic demands is in part fulfilled by autophagy^31,32^. Towards the end of the immune response, lymphocytes decrease their dependence on glycolysis, and cells selected for long-term immunological memory revert to OXPHOS fueled by fatty acid oxidation. Furthermore, memory B and T cells maintain a healthy mitochondrial pool to ensure energy supply without the harmful effects of oxidation^33^.

## Signaling nodes that integrate autophagy with the metabolic state

Cytoplasmic content subject to autophagic degradation in the lysosome is subsequently broken down into macromolecules that directly feed cellular metabolic pathways either to fuel anabolism or energy production. Furthermore, selective degradation of metabolic compartments such as mitochondria confers the cell with the capacity to rapidly redirect metabolite flow via autophagy. Thus, it is not surprising that signaling pathways that modulate autophagy also modulate cellular metabolism. MTOR and AMPK integrate environmental status, energy, and nutrient availability, coordinating metabolic pathways with autophagic activity.

The MTOR (mechanistic target of rapamycin kinase) pathway is an evolutionarily conserved nutrient-sensing pathway that plays broad roles in integrating the extracellular nutrient environment with the intracellular metabolic state. MTOR complex 1 (MTORC1) is a conserved serine/threonine kinase in the sensory module of this pathway and senses various stimuli, such as growth factors and nutrients, and directly orchestrates cellular decisions by modulating metabolism. The input-dependent activation of the MTOR networks is triggered by the phosphoinositide 3-kinase (PI3K)-AKT signaling axis, which promotes anabolism. In response to immunological cues such as those produced by the stimulation of CD28 or cytokines such as IL2 and IL4, the PI3K-AKT-MTOR axis upregulates the trafficking of SLC2A1/GLUT1 receptors to the cell surface, which drives glucose uptake and glycolysis and supports proliferation by generating essential metabolic intermediates. The MTOR pathway modulates the metabolic landscape through transcriptional reprogramming^34^. This reprogramming involves controlling transcription factors such as HIF1A, SREBF (sterol regulatory element-binding transcription factor), and MYC/c-Myc^35–38^. Furthermore, MTOR supports proliferation by enhancing protein production upon phosphorylation of EIF4EBP and RPS6KB/p70 S6 kinase, critical translation factors^39,40^. Several studies have implicated MTOR in integrating immunological stimuli and metabolic signals to coordinate an appropriate immune response by modulating immune cell function^41^.

Energy starvation triggers the activation of AMPK. When the ratio of ATP to AMP decreases, AMP stabilizes the phosphorylated form of AMPK and thus exerts an allosteric activating effect. ADP also stabilizes the phosphorylation status of AMPK; however, increased ADP levels may not trigger the allosteric change required for full AMPK activation^42,43^. ATP competes with AMP to bind to AMPK, and therefore, AMPK activation is highly responsive to the availability of cellular energy^44–48^. Furthermore, AMPK also responds to glucose availability and promotes OXPHOS by antagonizing MTOR in a cell-specific manner^49^. In summary, AMPK plays an important role in responding to glucose availability and maintaining immune homeostasis.

Autophagy is regulated by both MTORC1 and AMPK, which show reciprocal control through the regulation of the mammalian autophagy-initiating kinase ULK1. Under nutrient-rich conditions, ULK1 and ULK2 are phosphorylated by MTORC1, thus inhibiting autophagy. In addition, MTORC1 inhibits the PIK3C3/VSP34-containing class III phosphatidylinositol 3-kinase complex by phosphorylating ATG1 and AMBRA1^50^. In addition, MTORC1 modulates the transcriptional regulation of autophagy by phosphorylating and repressing TFEB^51^. In contrast, when there is a limitation of available nutrients, the inhibitory effect of MTORC1 on ULK1/2 is blocked. Furthermore, a decrease in the ATP:ADP/AMP ratio activates AMPK, which can directly activate ULK1/2 through phosphorylation^52^. Activation of ULK1/2 initiates autophagy and may play a key role in providing an alternate source of nutrients to promote immune cell responses under metabolic stress. Importantly, autophagy plays a critical role in lymphocyte development and function. Autophagy-deficient T and B cells fail to mature and show defective activation patterns. Below, we discuss this critical role of autophagy in conjunction with metabolic reprogramming in lymphocytes.

## T cells

The establishment and maintenance of an immune response and subsequent generation of memory are dependent on T cells. T cells coordinate adaptive immune responses such as the clearance or elimination of pathogens, tumor cells, and allergens. The role of cellular metabolism and autophagy in T cell function and differentiation has gained tremendous attention over the last decade. In this review, we consider how the cross talk of autophagy and metabolism influences T cell function, especially in the generation of immunological memory.

## T cell activation

Activated T cells must rapidly grow, divide, and exert effector functions. Therefore, upon activation, the bioenergetic requirements of T cells increase dramatically. T cells meet this demand by simultaneously increasing autophagy and glucose metabolism.

Autophagy plays a crucial role in the metabolic reprogramming of T cell survival^53^. Naïve CD4^+^ T cells use OXPHOS to meet their metabolic requirements. In addition, naïve T cells maintain basal levels of autophagy, and organelles such as mitochondria are the preferred cargo. Autophagy is rapidly induced in activated T cells^54^, which display an increase in autophagosome formation and clearance^55,56^. Following activation, organelles such as mitochondria are excluded from the autophagosome, suggesting that cargo selection is likely to be an activation-induced autophagic response in T cells^57,58^.

Several groups have generated autophagy-deficient mice and have shown that ablation of proteins in the core autophagy machinery (ATG3, ATG5, ATG7, ATG16L1, BECN1, or PIK3C3/VPS34) results in a significant reduction in thymocytes and peripheral T cells^54,56^. The decrease in the number of peripheral T cells may be a result of an increase in apoptotic rates, and several proapoptotic molecules, such as pro-CASP3 (caspase 3), CASP8, and CASP9, have been implicated in this process. In addition, organelle turnover, especially that of mitochondria and the endoplasmic reticulum, is affected in autophagy-deficient T cells. As a result, autophagy-defective T cells accumulate damaged mitochondria, which causes an increase in ROS production and consequently an increase in cell death. Together, the expression of proapoptotic factors and the increase in oxidative stress may contribute to the higher level of cell death that occurs in T cells that lack functional autophagy. Collectively, these studies elucidated the role of autophagy in modulating the homeostasis of T cells.

T cells exhibit a unique metabolic response: resting T cells prefer OXPHOS in the presence of oxygen, and newly activated T cells undergo glycolysis to meet their energetic demands. T cell activation causes a massive bioenergetic challenge, as the transition from naïve resting T cells to activated effector T cells involves extensive transcriptional and translational remodeling. These requirements result in complete metabolic reprogramming between the two subsets. To support these changes, T cell receptor (TR) engagement induces not only autophagy but also several nutrient-sensing/response pathways, such as those involving MTOR. Upon receiving signaling via IL7 and TR, these cells maintain their mitochondrial potential and ATP synthesis through a steady influx of glucose enabled by the expression of SLC2A1/GLUT1 in a PI3K-AKT-dependent manner, which in turn activates MTOR^59^. MTOR activation results in a global change in the metabolic transcriptome with the induction of MYC and HIF1A. These transcription factors regulate glucose transporters and glycolytic enzymes, which, in turn, switch the metabolism required for naïve T cells, which predominantly undergo OXPHOS, that required for activated T cells, which is glycolysis^38^. Corroborating this observation, autophagy-deficient T cells show decreased ATP production in response to TR engagement, and both glycolysis and mitochondrial respiration decrease as a result of inhibited autophagic activity^53^.

## T cell differentiation

A critical characteristic that defines immune functionality and longevity in T cells is the degree of differentiation of specialized subpopulations. CD4^+^ T helper (Th) cells modulate the immune response by producing cytokines that enhance or suppress inflammation, and CD8^+^ cytotoxic T cells kill pathogen-infected or malignant cells by secreting inflammatory cytokines and cytolytic molecules^60^. Upon activation, T cells grow, proliferate, and ultimately differentiate into distinct T cell subsets that exhibit different functional responses. The heterogeneity of T cell fate in various effector subsets is regulated by lineage-specific convergence of autophagy and metabolism^33^. The biochemical demands of energy/metabolic intermediates are not uniform across the various subsets; therefore, cell type-specific metabolic programs are upregulated upon activation to support the differentiation of T cells into their separate lineages.

### CD4^+^ T cell differentiation and immune suppression

The differentiation of CD4^+^ T cells in response to specific cytokine signals into T helper cells (Th1, Th2, Th9, Th17, and follicular T cells) or regulatory T cells (Tregs) is accompanied by the modulation of their cellular metabolism and autophagy to meet their bioenergetic requirements. The distinction of these subsets at the metabolic level was determined when the role of MTORC1 in T cell differentiation was investigated. Although MTOR-deficient T cells proliferate and survive, their differentiation program is skewed towards the Treg lineage^61^. Consistently, pharmacological ablation of MTOR activity compromises the differentiation of T helper cells, specifically Th1, Th2, and Th17 cells, and favors Tregs^62–65^. In addition, MTORC1 plays an important part in dictating T cell fate, as it plays a critical role in establishing the glycolytic metabolic state and thus T helper cell identity. T helper cells favor glycolysis and express high levels of SLC2A1/GLUT1, whereas Tregs preferentially undergo AMPK-driven fatty acid oxidation for energy production. Consistently, Tregs show increased levels of AMPK activation, and pharmacological stimulation of AMPK activity in vivo increases the number of Tregs^61^. Transcription factors such as HIF1A and FOXP3 further support the establishment of the metabolic state and T cell identity of T effector and Treg cells, respectively^66^.

Regulatory T cells constitute a population of suppressor CD4^+^ T cells that modulate the activity of the immune system. These cells control the immune response to self-antigens and thus prevent autoimmune disease. From a metabolic viewpoint, resting T cells are relatively inert and require low amounts of energy compared to activated Tregs. To meet the bioenergetic expenditure of activation, resting T cells utilize various substrates, such as glucose, amino acids (in particular, glutamine), and fatty acids. Thus, Treg cells rely on OXPHOS and lipid metabolism for survival. The distinct signaling pathways and metabolic preferences of Tregs drive their function and enable their survival, distinguishing them from other T cell subsets. Tregs expressing coreceptor CD4, cytokine receptor IL2RA/CD25 and transcription factor FOXP3 represent the best-characterized subset of Tregs. FOXP3 reprograms T cell metabolism by suppressing glycolysis and enhancing OXPHOS. This shift is further supported by MTORC1, which supports lineage commitment and suppresses functions via metabolic reprogramming. RPTOR/raptor, an essential component of the MTORC1 complex, promotes mevalonate pathway activation to coordinate Treg proliferation and the production of the important suppressive molecules CTLA4 and ICOS.

The view that glycolysis, while required to achieve functional competency, is critical for facilitating the enhanced proliferation of activated T effector cells is changing, as studies using nonfermentable carbon sources show that T cells successfully rely on OXPHOS for proliferation and survival. However, under OXPHOS, T cell effector function is compromised, as shown in their inability to synthesize IFNG/IFNγ or IL2. This defect lies at the level of mRNA translation. Engagement of aerobic glycolysis increases the association of *IFNG* mRNA with translation factors, thus enabling its efficient translation. In the absence of aerobic glycolysis, the defective translation of IFNG is due to the enhanced binding of GAPDH (a key glycolytic enzyme) to *IFNG* mRNA. Therefore, by engaging in aerobic glycolysis, T cells disrupt the GAPDH and *IFNG* mRNA interaction, thus allowing efficient translation of IFNG. Another glycolytic enzyme with additional effects that regulate T cell function is LDHA (lactate dehydrogenase A)^67^. LDH converts pyruvate to lactate and balances the reductive capacity of the cell by regenerating NAD^+^. LDHA and LDHB are expressed as five tetrameric LDH isoenzymes; however, LDHA is induced in T cells upon activation via HIF1A- and MYC-induced transcription^38^. Whereas the expression of this enzyme supports glycolysis, it independently promotes the expression of IFNG via an epigenetic mechanism. LDHA enhances histone acetylation and thus transcription of *IFNG* by maintaining high levels of acetyl-coenzyme A (CoA)^67^. Biochemically, acetyl-CoA is enhanced in the cell when pyruvate is redirected to the TCA cycle, and then, citrate is exported from mitochondria and used in the generation of acetyl-CoA. Subsequently, this acetyl-CoA can be used to promote histone acetylation at specific gene loci. Therefore, functional aerobic glycolysis in activated T cells is necessary to fully realize their effector function. Importantly, these studies highlight the role of mitochondrial metabolism during effector T cell differentiation. Bailis et al. showed that the differentiation and terminal effector functions of Th cells are biochemically uncoupled at the mitochondria^68^. Using genetic and pharmacological inhibition, they showed that complex 1 and complex 2 of the electron transport chain perform distinct roles to drive T cell differentiation and effector function. Specifically, complex 1 is crucial for the generation of the metabolite intermediates required for histone acetylation, such as acetyl-CoA, by enhanced mitochondrial citrate export. This process promotes Th cell proliferation. However, complex 2 restricts mitochondrial citrate export by promoting the movement of carbon in the TCA cycle and therefore helps in establishing the terminal effector state of Th1 cells. In summary, this biochemical intermediate represents a regulatory module that works in parallel with transcriptional reprogramming to drive T cell differentiation^68^.

In a model of murine inflammatory bowel disease, loss of autophagy alters the number of CD4^+^ T cells in the gut. Ablation of ATG16L1 results in the reduction of the Treg subpopulation but the expansion of the Th2 cell subpopulation in peripheral tissues^69^. The different survival rates of the different subsets of CD4^+^ T cells are associated with autophagy-mediated alterations in their metabolic profiles. Tregs rely on mitochondrial respiration for ATP production. However, loss of autophagy results in enhanced expression of glycolytic genes and MTOR activation, thereby switching the metabolic state to the acquisition of the glycolytic phenotype. Thus, autophagy is important to maintain the functional integrity of Tregs. Interestingly, loss of autophagy has the opposite effect on Th2 cells. Th2 cells maintain high levels of MYC and glycolytic gene expression. Therefore, glucose metabolism is not altered in these cells irrespective of *ATG16L1* gene deletion. Thus, the glycolytic phenotype of Th2 cells might better equip these cells to adapt to the metabolic switch towards glycolysis induced by autophagy deficiency. In addition, ATG7-deficient Treg cells lose their oxidative phenotype and display an increase in glycolytic activity upon TR engagement^70^. The authors showed that ATG7-deficient Tregs have altered transcriptional programs with high MTORC1 activity and MYC expression. These results indicate that autophagy is critical in regulating Treg metabolism by controlling transcriptional programs in the cell^70^. Importantly, autophagy is required for the suppressor function of Tregs. Autophagy also helps in the survival of Tregs during growth factor withdrawal. In contrast, induction of autophagy in T effector cells leads to cell death. This difference shows the remarkable versatility of the process and adaptation to changing microenvironments. Tregs must travel to places in the body that are deprived of nutrients and, hence, autophagy acts as a salvage pathway to restore the internal nutrient pool. Therefore, autophagy couples environmental cues and metabolic homeostasis to promote the survival of Tregs.

### CD8^+^ T cell differentiation and immunological memory

Resting CD8^+^ T cells, similar to CD4^+^ T cells, undergo dynamic changes in the reprogramming of their cellular metabolism from oxidative respiration to aerobic glycolysis upon activation. This switch is essential to support the clonal expansion of cytotoxic T cells, which are highly proliferative and produce inflammatory cytokines and cytolytic granules such as PRF1 (perforin 1) and GZMB (granzyme B). Following antigen removal, a large population of cytotoxic T cells undergoes apoptosis, while a small subset differentiates into long-lived quiescent memory CD8^+^ T cells. Memory CD8^+^ T cells revert to resting T cells with OXPHOS metabolism as they no longer undergo rapid proliferation.

As with CD4^+^ T cells, CD8^+^ T cells induce glycolysis upon TR engagement. Interestingly, Menk et al. showed that this induction of glycolytic flux is not controlled by increased glucose uptake and occurs in a transcription- and translation-independent manner^71^. Furthermore, the signaling cascade that initiates glycolysis immediately after T cell activation is independent of CD28 and AKT signaling. Consistent with this finding, pharmacological and genetic ablation of AKT kinase showed that this pathway is dispensable for activation-induced glycolysis in murine effector CD8^+^ T cells. Notably, the authors determined that the initiation of aerobic glycolysis is mediated by PDH (pyruvate dehydrogenase) and is required for optimal cytokine production and not for proliferation or cytolytic function. They determined that the cytokines IFNG, IL2, and TNF are under the control of PDH-mediated regulation. Interestingly, LDH, similar to GAPDH, binds to cytokine mRNA, thus sequestering it away from the translational machinery^72^. This mechanism, however, is opposite that is shown in CD4^+^ T cells, wherein LDH activity is independent of 3′ UTR regulation.

Memory CD8^+^ T cells are a long-lived population of antigen-specific T cells that provide an enhanced protective response to secondary infection with a previously encountered antigen. These cells persist for decades in the body without antigen stimulation; therefore, the maintenance of their functionality requires active programming of both cellular metabolism and clearance of cytoplasmic debris. Similar to resting T cells and CD4^+^ Tregs, memory T cells favor mitochondrial respiration, specifically, lipid oxidation. Consistently, these cells show a high level of mitochondrial spare respiratory capacity (SRC), which is extra capacity available to produce energy in response to increased metabolic stress^73^.

Several studies have shown that autophagy is a key regulator of memory T cell generation and maintenance. In studying a mouse model of acute infection with lymphocytic choriomeningitis virus, key autophagy genes, such as *Atg5* or *Atg7*, were ablated in CD8^+^ T cells following antigen presentation, and the development of memory CD8^+^ T cells was assessed^74^. Whereas the ablation of these genes has no effect on the proliferation or function of effector cells, ATG7-deficient CD8^+^ T cells show survival defects during the effector-to-memory transition and exhibit defects in the development of long-term memory T cells. Furthermore, ATG7-deficient CD8^+^ T cells show drastic changes in their transcriptional and metabolic profiles, suggesting that autophagy supports metabolic homeostasis during the development of memory T cells from effector T cells. In contrast, in a tumor microenvironment, ATG5-deficient CD8^+^ T cells show a dramatic shift in their differentiation and acquisition of an effector memory phenotype and produce high amounts of TNF and IFNG^75^. Interestingly, these cells show an increase in glucose metabolism and a reduction in internal S-adenosyl methionine levels. Furthermore, these cells show alternate histone methylation and global transcriptional changes favoring a glycolytic effector memory T cell phenotype. These studies highlight the role of autophagy in favoring different metabolic states across different T cell subsets in a context-dependent manner. In addition, autophagy helps to accommodate bioenergetic demands by cooperating with metabolic pathways and epigenetic modifications to meet the biochemical demands of T cell activation. Therefore, the cellular fate of T cells is determined by the regulation of autophagy and the subsequent control of metabolic activity.

## B cells

B cells are critical for mediating humoral immunity, which involves the production of antibodies that recognize antigens. Antibodies have unique antigen-binding pockets and reside on the membrane of B cells before secretion, serving as part of the BCR. While few studies have examined B cells in the context of autophagy and metabolism, it is speculated that the cross-talk between autophagy and metabolism pathways serves a regulatory function similar to that in T cells.

## B cell development

In mammals, B cells arise from a common lymphoid progenitor and develop in the bone marrow. Pro-B cells represent the earliest stage of B cell development and are characterized by a quiescent metabolic state^76^. The transition of pro-B cells to pre-B cells is marked by the rearrangement of the immunoglobulin heavy chain loci. Signaling cues emitted from pre-BCRs expressed on the cell surface rapidly induce clonal proliferation and the recombination of immunoglobulin light chain genes^77^. In-frame IgG gene rearrangements in small pre-B cells subsequently result in the expression of a BCR on immature B cells. Upon selection, these immature B cells form the pool of long-lived immature B cells.

The development of pro-B cells into small pre-B cells is driven by metabolic changes induced by pre-BCR signaling. Large B cells internalize high levels of glucose compared to pro-B and small pre-B cells. EFHD1/Swiprosin-2, an inner mitochondrial membrane protein, regulates the metabolic switch critical for the transition of pro-B cells into pre-B cells. EFHD1 is expressed in pro-B cells and maintains oxidative respiration in these cells. In large pre-B cells, the expression of the pre-BCR downregulates EFHD1, resulting in an increase in glycolysis in these cells. When large pre-B cells transition to small pre-B cells, pre-BCRs are lost, and the metabolic state transitions to OXPHOS. Upon deletion of *EFHD1*, the metabolism of pro-B cells switches to a more glycolytic phenotype with an increase in basal glycolysis, maximum glycolytic capacity and glycolytic reserves, and a decrease in the oxygen consumption rate (OCR):extracellular acidification rate (ECAR) ratio. Thus, the switch to glycolysis in large pre-B cells increases their metabolic activity to meet their high energy requirements and to fuel anabolism^78^.

The role of autophagy in B cell development is controversial. In ATG5-deficient mice generated by transferring *atg5*^−*/*−^ fetal hematopoietic cells into lethally irradiated wild-type congenic hosts, the lymphocyte population, including both B and T cells, is reduced^56^. This finding suggests that autophagy may play a role in B-cell development. Specifically, *atg5*^−*/*−^ progenitors display a severe defect in the transition from the pro- to pre-B cell stage in bone marrow^56,79^. However, in these studies, the fetal liver chimeras used to generate B cells were completely devoid of the *Atg5* gene, causing speculation that the block in development may have been due to early defects in hematopoietic development. Studies using mouse models with conditional deletion of *Atg5* under developmental promoters showed that whereas the basal level of autophagy is necessary for mature B cell survival, it is not required for the transition between pro- and pre-B cell stages^80^. However, whether the requirement for autophagy can be uncoupled during the process from the transition state to the survival of B cells remains to be explored.

## B cell activation

Small pre-B cells in the bone marrow migrate to the spleen, where they further mature into follicular B cells (circulating) or marginal zone B cells (noncirculating). Their fate is driven by the strength of BCR signaling^76,81^. B cells produce antibodies in response to the binding of an antigen to BCRs. Whereas this engagement alone initiates a cascade of signaling events, it is not enough to fully activate B cells. Activation requires a second, temporally distinct signal provided by antigen-specific helper T cells following processing and presentation of the antigen by B cells^81,82^. In the absence of T helper cells, the second signal requirement can be fulfilled by pattern recognition receptors. This two-pronged control mechanism not only prevents the proliferation and differentiation of B cells into antibody-secreting cells in the absence of antigen but also initiates the metabolic reprogramming required for B cell activation^83,84^. In addition, this dual programming equips the B cell precursors with the metabolic machinery needed to survive and differentiate following activation. B cells transition from OXPHOS to glycolysis with an increase in SLC2A1/GLUT1 expression and glucose uptake. Interestingly, the mitochondrial mass and the rate of OXPHOS, as measured by OCR, also increases in B cells following antigen stimulation. Another measure that reflects the metabolic switch is the ratio of OCR to ECAR. Activated T cells exhibit a lower OCR:ECAR ratio than naïve T cells. In contrast, this ratio remains unchanged in B cells after stimulation, suggesting metabolic reprogramming that results in a balanced increase in both glycolytic and mitochondrial activity in activated B cells^85^.

Upon activation, similar to T cells, B cells undergo transcriptional and metabolic remodeling that primes the cells for rapid proliferation and growth in regions called germinal centers (GCs)^84,86^. GCs are dynamic anatomical structures where B cells undergo affinity maturation through somatic hypermutation and, infrequently, class switching recombination supported by their unique microenvironment. GCs consist of two histologically distinct regions: the dark zone and the light zone. Highly proliferative GC B cells undergo class switching recombination and somatic hypermutation in the dark zone, whereas nondividing GC B cells present cognate antigens. The GC B-cell stage represents the transition period where the fate of the B cell is determined. Transcriptional programs initiated in B cells determine whether B cells differentiate into memory B cells or plasma cells. Therefore, these cells are under massive bioenergetic pressure to differentiate^87^. To relieve this bioenergetic pressure, the metabolic activity of GC B cells is fueled by enhanced glucose uptake and mitochondrial biogenesis^84^. Multiple studies have reported that GC B cells show enhance glycolysis that supports anabolism in hypoxic microenvironments^88^. Notably, GSK3 is a metabolic sensor that promotes the growth and proliferation of B cells by enhancing glycolytic activity. Furthermore, GSK3 prevents ROS-induced apoptosis of GC B cells in the highly proliferative GC microenvironment^89^. However, this view was recently challenged by Weisel et al., who demonstrated that GC B cells primarily utilize fatty acid oxidation to meet their metabolic demands^90^. In any case, the metabolic heterogeneity of B cells in GCs needs to be carefully assessed, as glycolysis may be limited to the initial phase of clonal expansion when an energy boost is required.

GC B cells, along with metabolic reprogramming, recycle their own mass via autophagy^75^. Noncanonical autophagy has been implicated in the early stages of GC B cell activation involving WIPI2^91^. Whereas antigen stimulation via the BCR or TR increases MTOR activity, which subsequently decreases canonical autophagy, GC B cells temporarily switch to noncanonical autophagy to meet their metabolic demands. Noncanonical autophagy, in this context, is defined as the induction of autophagy that is accompanied by the activation of MTOR, as measured by phosphorylation of RPS6KB/p70 S6 kinase. Upon genetic ablation of WIPI2 in B cells, the balance of canonical and noncanonical autophagy is shifted. Interestingly, ablation of ATG16L1 does not result in any defects. Furthermore, this results in the perturbation of metabolic status and mitochondrial homeostasis. These results suggest an elegant regulation that enhances proliferation via MTOR activity and provides the necessary intermediates required for proliferation by metabolic reprogramming and autophagy^91^.

## B cell differentiation

Following antigen presentation, a subset of B cells survives the rapid proliferative phase known as the GC reaction. These cells differentiate into either plasma cells or memory B cells. Here, we discuss several studies that have reported the crucial role of autophagy and metabolic regulation in modulating the function and survival of plasma cells and memory B cells.

### Plasma cells

Plasma cells are antibody-producing cells that form a heterogeneous population differing in lifespan, ontogeny, and anatomical function. The identity of plasma cells is established by extensive transcriptional reprogramming^92^. Importantly, IRF4 and PRDM1/Blimp-1 are responsible for establishing plasma cell identity^93,94^. This program primes the cell for robust production of secretory immunoglobulins by expanding the ER and enhancing its protein folding capacity^94^. To manage the assembly and trafficking of immunoglobulin cargo, plasma cells need to efficiently manage the supply of metabolites for synthesis and employ adaptive strategies to curtail the stress resulting from this overload. Therefore, plasma cells reprogram their metabolism to meet the demands of antibody production and induce autophagy to counteract ER stress.

In response to infection, GC B cells are activated through T cell-dependent reactions to become antibody-producing plasma cells. Over time, these reactions affecting GC B cells produce plasma cells of longer lifespans encoding higher-affinity antibodies. A subset of long-lived plasma cells (LLPCs) can also be produced in a T cell- and GC-independent manner^95^. TLR stimulation produces a type of humoral response that is also T cell-independent and GC independent, in which extrafollicular short-lived plasma cells are generated as an early response to provide partial control of infection. Interestingly, the lifespan of plasma cells is associated with antibody secretion, autophagy, and nutrient uptake^96,97^.

When B cells are activated under plasma cell differentiating conditions, they undergo a proportional increase in glycolysis and OXPHOS^98^. This change suggests the high degree of metabolic flexibility in activated B cells that allows them to channel metabolic substrates and ATP into various processes. LLPCs exhibit increased glucose uptake compared to short-lived plasma cells. Consistently, the former also show high expression of SLC2A1/GLUT1. LLPCs predominantly rely on glucose for antibody glycosylation. However, similar to T cells, glucose metabolism via glycolysis is necessary for B cells to have fully realized functionality. Inhibition of glycolysis in B cells compromises the secretion of IgG and IgM antibodies in response to lipopolysaccharide (LPS) stimulation^85^. Glucose metabolism generates acetyl-CoA, which can be channeled into both fatty acid and mevalonate synthesis pathways. Fatty acids are crucial metabolic intermediates for the synthesis of phospholipids and protein modification. Plasma cells are characterized by high rates of protein synthesis and expansion of the ER. Therefore, they have a high demand for lipids and cholesterol to support the increase in membrane biogenesis associated with ER expansion and proliferation. In response to LPS, plasma cells reprogram glucose utilization to promote de novo lipogenesis^99^.

ACLY (ATP citrate lyase) is a rate-limiting enzyme that produces cytosolic acetyl-CoA from mitochondrial citrate and is a crucial link between glucose metabolism and fatty acid synthesis. Pyruvate from glucose metabolism is imported into the mitochondria, where it is converted to citrate. Citrate can be exported from mitochondria via the malate citrate shuttle and can be used as a substrate for ACLY to catalyze the formation of acetyl-CoA^100^. Ablation of ACLY not only reduces glucose-dependent de novo fatty acid synthesis but also perturbs LPS-induced changes that induce the acquisition of a plasma-like B lymphocyte phenotype. Therefore, ACLY represents an important metabolic checkpoint for plasma cell differentiation in response to TLR stimulation^99^. Consistently, LLPCs show increased expression of MPC2, an essential component of the mitochondrial pyruvate carrier, and enhance mitochondrial pyruvate in response to immunization. In this way, the utilization of glucose is programmed in a cell subset-specific manner^96^.

Several reports have highlighted the role of autophagy in maintaining the plasma cell population^101,102^. Plasma cells show high autophagic activity with an increase in GFP-LC3 puncta in long-lived bone marrow plasma cells. When ATG5-deficient murine B cells are activated ex vivo with LPS stimulation, differentiated plasma cells display higher ER content and ER proteins. In addition, these cells display a defective immunoglobulin response to antigens. Further analysis demonstrated that the specific degradation of the ER by autophagic mechanisms in plasma cells is necessary to control ER homeostasis and the function of differentiating plasma cells. In addition, these cells exhibit high levels of ER stress, which, interestingly, augments the expression of PRDM1 and immunoglobulins. This finding suggests that autophagy exerts a negative control on ER capacity and immunoglobulin synthesis. In addition, autophagy supports plasma cell maintenance by sustaining energy production during antibody secretion, which is associated with high metabolic demand. Notably, ATP production in autophagy-deficient B cells is compromised, making the cells more susceptible to apoptosis. Therefore, autophagy plays the dual role of generating metabolic substrates and controlling immunoglobulin production to maintain plasma cell viability^102^. Although the role of autophagy in plasma cell maintenance is clear, whether autophagy is necessary for plasma cell differentiation remains controversial and requires further study.

### Memory cells

A subset of B cells is chosen to survive and exit the GC, and these cells ultimately differentiate into long-lived antigen-experienced cells known as memory B cells. These memory B cells represent humoral immunological memory, and they rapidly expand and differentiate into plasma cells upon secondary antigen challenge. Memory B cells, similar to naïve cells, are quiescent before differentiation into plasma cells when stimulated by antigen. However, memory B cells have lower thresholds for activation and greater proliferative capacity than naïve cells. Importantly, memory B cells survive for decades even in the absence of antigen stimulation, thus providing long-lasting protection against secondary infections. The quiescent state of memory B cells is achieved by tightly regulated suppression of the cell cycle and differentiation programs. Therefore, memory B cells can modulate their metabolic state, maintain their quiescent state and prolong their survival. However, only a few studies have investigated the role of autophagy and metabolism in the maintenance of memory B cells.

In a model of influenza infection, deletion of *Atg7* in B cells leads to the loss of memory B cells and secondary antibody responses^103^. Furthermore, these cells display reduced mitochondrial membrane potential and increased ROS production, suggesting that autophagy is crucial for the maintenance of mitochondrial homeostasis in memory B cells. Autophagy-mediated clearance of mitochondria can eliminate the accumulation of damaged mitochondria and, subsequently, the accumulation of death-promoting molecules. Consistently, proteins involved in mitochondrial apoptosis have been implicated in memory B cell survival. However, the mechanisms, specifically the role of mitophagy and metabolic regulators, that mediate the long-term survival of memory B cells remain to be elucidated.

## Concluding remarks

We are just beginning to dissect the intricate molecular details intertwined in autophagy and metabolism. The possibility of pharmacological targeting of these pathways to influence specific aspects of immune cell behavior will prove to be beneficial in treating diseases such as cancer. The interplay between autophagy and metabolism in T lymphocytes is of interest due to the capacity of this population to engage the immune system in fighting against cancer. Persistent antigen exposure in tumors accelerates T cell exhaustion; however, certain populations of tumor-infiltrating T cells (TILs) maintain stemness and preserve tumor clearance capabilities. The capacity to preserve the stemness of these cells is leveraged to engage the immune system in fighting against cancer via immunotherapy strategies such as checkpoint blockade and adoptive cellular therapy. However, the underlying mechanisms involved in TIL generation and maintenance are unknown^104^. Recently, Vodnala and colleagues revealed the role of autophagy in maintaining TIL stemness^105^. They showed that epigenetic regulation of TIL stemness is governed by metabolism, specifically, the depletion of intracellular acetyl-CoA and methionine. Elevated levels of potassium in the tumor microenvironment cause a starvation response, which induces autophagy, mitochondrially dominant metabolism, and an insufficiency of the epigenetic remodelers required for differentiation^105^. Interestingly, they showed that induction of autophagy in T cells directly by pharmacological activation or by gene therapy or indirectly by elevating potassium concentration increases the retention of T cell stemness. Therefore, autophagy and metabolism can be manipulated to enhance anticancer therapeutic strategies, especially checkpoint blockade, to induce the stemness of antitumor T cells. Thus, cross-talk between autophagy and metabolism pathways can have implications with regard to T cell function and, therefore, is an important consideration for enhancing the efficiency of immunotherapies using adaptive immune cells.

A current bottleneck in exploring the effect of autophagy and metabolism in immune subtypes is the use of techniques that assess functions through the use of bulk assays. The cellular metabolic state is elucidated by quantifying metabolite abundance by mass spectrometry or by measuring oxygen consumption and acidification of external media as a proxy for OXPHOS and glycolysis, respectively. Similarly, assessment of autophagy activity has historically involved bulk cell populations. While these techniques have provided invaluable insights, they fall short in answering questions regarding metabolic heterogeneity and fail to provide a more comprehensive understanding of the interplay between the metabolic pathway and cellular processes that dictate immune cell function. Further complications arise when working with sparse human clinical material where tissue-specific data are necessary to determine the potential implication in human diseases. Recently, several studies have provided insights into technological solutions to assess metabolic states and autophagy at the single-cell level^106–108^. These approaches enable the study of the cellular metabolic state and autophagy activity in conjunction with cellular identity. In the context of cancer, this approach might prove to be particularly useful in understanding how clonal subsets display differential autophagic activity upon chemotherapy treatment.

**Fig. 1:**
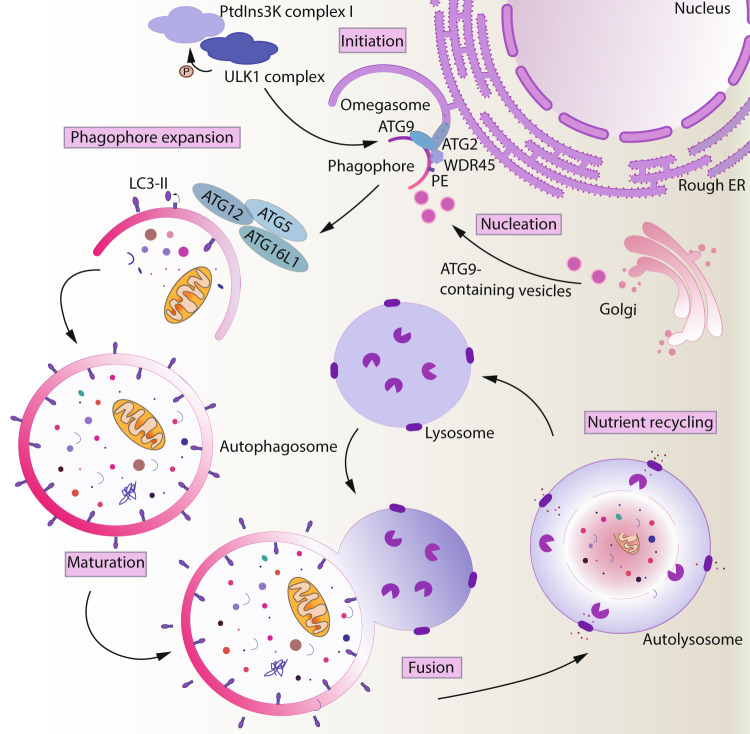
Overview of autophagy. Macroautophagy is initiated by the ULK1 and PIK3C3/Vps34 complexes, which trigger the nucleation of the phagophore at the omegasome. The membrane sources required for phagophore biogenesis are supplied by ATG9-containing vesicles and phospholipids channeled from the ER into the growing phagophore by the ATG2-WDR45/WIPI4 complex. Two ubiquitin-like conjugation systems facilitate phagophore expansion and closure. Subsequently, the autophagosome fuses with the lysosome, where its contents are degraded, and the macromolecules thus generated are recycled.

Adaptive immune cells can rapidly transition between quiescent and proliferating states to mount an immune response. Studies from the last decade have established that the diverse capabilities of these immune cells are enabled by the coordination between cellular metabolism and autophagy in a context-dependent manner. This review has summarized studies highlighting the molecular events that control the intertwining of autophagy and metabolism during important intracellular events such as development and differentiation. Multiple signaling pathways converge to modulate autophagy and metabolism at the level of transcription, posttranscription, and translation. Although the hierarchy of signaling relays is not clearly understood, this regulatory landscape is affected by feedback from external nutrient cues, the internal metabolic state, and cellular identity. Therefore, we emphasize that the coordination of metabolism and autophagy in T and B cells is highly context dependent (Fig. 1).
